# Supplementary figures and images for: Potential urinary aging markers of 20-month-old rats
Source: PeerJ. 2016 Jun 7;4:e2058. doi: 10.7717/peerj.2058 (PMC4906655; doi:10.7717/peerj.2058)

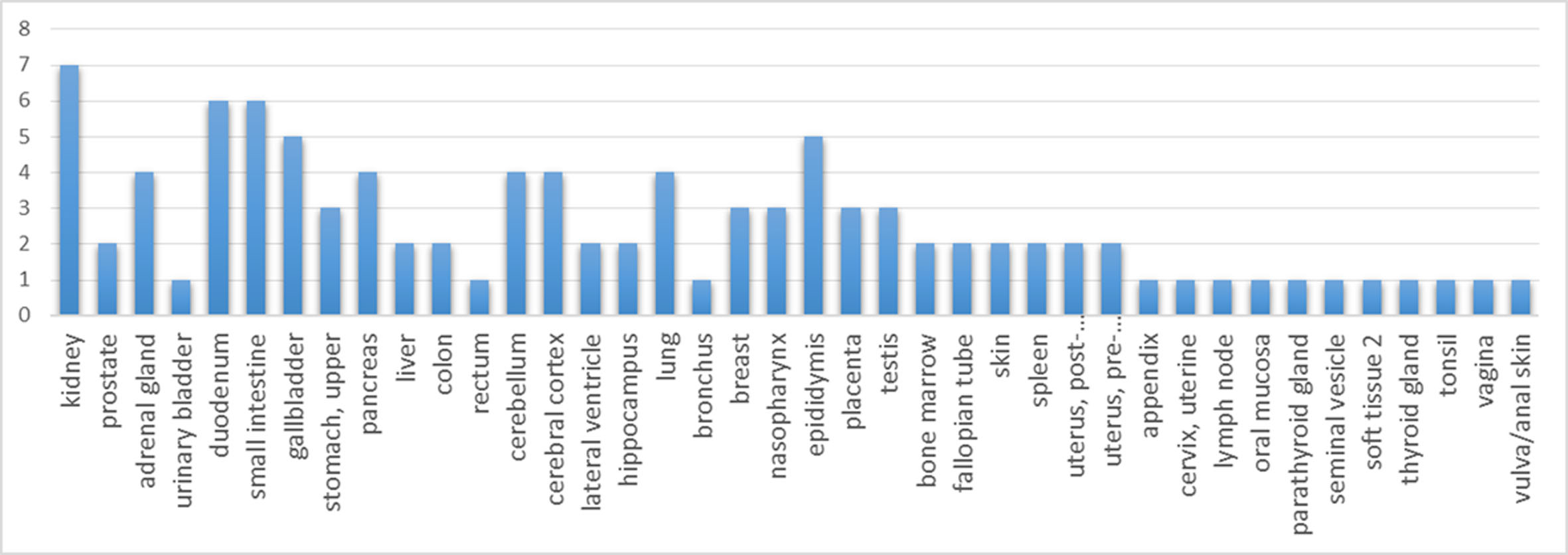

Supplement: Figure S1 [file peerj-04-2058-s003.jpg]
